# The β-lactam adjuvant guanosine potentiates anti-folate antibiotics and pyrimidine synthesis inhibitors by depleting thymidine in methicillin-resistant *Staphylococcus aureus*

**DOI:** 10.1128/aac.00377-26

**Published:** 2026-06-10

**Authors:** Aaron C. Nolan, Jessica B. Kelly, Jongsam Ahn, Dhananjay Shinde, Vinai C. Thomas, Merve S. Zeden, James P. O'Gara

**Affiliations:** 1 Microbiology, School of Biological and Chemical Sciences and Institute for Health Discovery and Innovation, University of Galway 8799, Galway, Ireland; 2 Department of Pathology, Microbiology and Immunology, University of Nebraska Medical Center 198515, Omaha, Nebraska, USA; The Peter Doherty Institute for Infection and Immunity, Melbourne, Victoria, Australia

**Keywords:** *Staphylococcus aureus*, MRSA, nucleotide metabolism, thymidine, anti-folate antibiotics, β-lactam resistance, pyrimidine antimetabolites, anti-cancer drugs, ROS

## Abstract

Efforts to improve the effectiveness of existing interventions for antimicrobial-resistant infections include identifying new ways to overcome resistance to licensed antibiotics using adjuvants or deploying antibiotics in novel combinations. Although antibiotics targeting the bacterial cell wall (e.g., β-lactams) and folate metabolism (e.g., trimethoprim-sulfamethoxazole [TMP-SMX]) remain cornerstones of modern healthcare, resistance to both classes poses an ongoing therapeutic challenge. We recently demonstrated that purine nucleosides can act as potent antibiotic adjuvants, restoring β-lactam susceptibility in methicillin-resistant *Staphylococcus aureus* (MRSA). Here, we show that guanosine significantly reduces intracellular thymidine levels in MRSA and potentiates the activity of antifolate antibiotics (TMP-SMX), as well as the pyrimidine antimetabolites 5-fluorouracil and 5-fluorouridine. Incorporation of oxacillin into guanosine-antifolate or guanosine-pyrimidine analog combinations further enhanced killing of both planktonic and biofilm-associated MRSA. Thymidine depletion was accompanied by elevated intracellular reactive oxygen species and dissipation of membrane potential, providing mechanistic insight into the bactericidal effects of these combinations. Together, these findings demonstrate that guanosine expands MRSA susceptibility beyond β-lactams to include additional clinically relevant antimicrobial drug classes commonly used to treat bacterial infections.

## INTRODUCTION

Finding new ways to preserve the efficacy of currently licensed antimicrobial drugs is a central part of efforts to address the antimicrobial resistance (AMR) crisis. In addition to new drug discovery, which is costly and time-consuming, repurposing currently licensed antibiotics in new combinations, and the discovery of novel adjuvants also have significant potential to improve treatment options for AMR infections. Antibiotic adjuvants, which potentiate antibiotic activity or disrupt resistance mechanisms, have proven clinical usefulness. In 2023, we reported that the purine nucleosides guanosine (Gua) and xanthosine (Xan) have activity as adjuvants that can resensitize methicillin-resistant *Staphylococcus aureus* (MRSA) to oxacillin (OX) and other β-lactam antibiotics (1). Exposure of MRSA to Gua significantly reduced the levels of the cyclic dinucleotide c-di-AMP, which is required for β-lactam resistance (1). In contrast, exposure of MRSA to adenosine (Ade) did not significantly affect c-di-AMP levels and instead increased OX resistance. Inosine, which can be fluxed into the ATP or GTP branches of purine metabolism, only marginally affected OX resistance (1). c-di-AMP controls osmotic regulation, and exposure of MRSA to OX/Gua was accompanied by a twofold increase in cell size (1). These data support the conclusion that perturbation of purine homeostasis by exogenous nucleosides controls MRSA resistance to β-lactams. Drugs derived from nucleotides are used in the treatment of cancer and viral infections, raising the possibility that purine nucleosides may potentiate the activity of β-lactams against MRSA.

In this work, we compared the impact of exposure to OX/Gua or OX/Ade combinations on the MRSA metabolome and revealed significant pleiotropic effects on cell wall, purine, and pyrimidine (specifically thymidine) metabolism. Thymidine biosynthesis is dependent on tetrahydrofolate (THF), which serves as a co-factor for thymidylate synthase (ThyA)-mediated conversion of uracil into thymidine (2–4), suggesting that purine adjuvants may potentiate the activity of the anti-folate antibiotics trimethoprim-sulfamethoxazole (TMP-SMX), as well as the pyrimidine analog anti-cancer drugs 5-fluorouracil (5-FU) and 5-fluorouridine (5-FUrd). TMP-SMX, which is used in a 1:5 ratio combination, blocks the activity of dihydrofolate reductase and dihydropteroate synthase, respectively, required for THF synthesis. This formulation, also known as bactrim and co-trimoxazole, is commonly used to treat MRSA (5) and several other bacterial infections (6). However, resistance is observed in the clinic, especially in chronic *S. aureus* infections with the rise of thymidine-dependent small colony variants, with mutations in *thyA* (2–4, 7, 8).

Reduced thymidine levels in OX/Gua-treated MRSA raised the possibility that purine adjuvants may also potentiate the activity of antibiotics that interfere with thymidine and/or pyrimidine biosynthesis. To investigate this, synergy between Gua and the anti-folate antibiotics sulfamethoxazole (SMX), trimethoprim (TMP), and the pyrimidine analogs 5-FU and 5-FUrd was measured. The activity of these antibiotic/adjuvant combinations against MRSA growing planktonically and in biofilms was investigated and expanded to include OX. Finally, the impact of reduced thymidine levels on the accumulation of reactive oxygen species (ROS) and oxidative stress-related membrane potential was investigated to gain further mechanistic insights. Our data reveal that the purine nucleoside adjuvant Gua also potentiates the activity of anti-folate antibiotics and pyrimidine analogs against MRSA. Furthermore, enhanced adjuvant activity was measured when OX was included in these antibiotic/adjuvant combinations. These data further support the potential of Gua as an antibiotic adjuvant, enhancing the activity of two separate classes of clinically used antibiotics against MRSA.

## RESULTS

### The purine adjuvant guanosine downregulates thymidine levels, revealing a new therapeutic target

**Fig 1:**
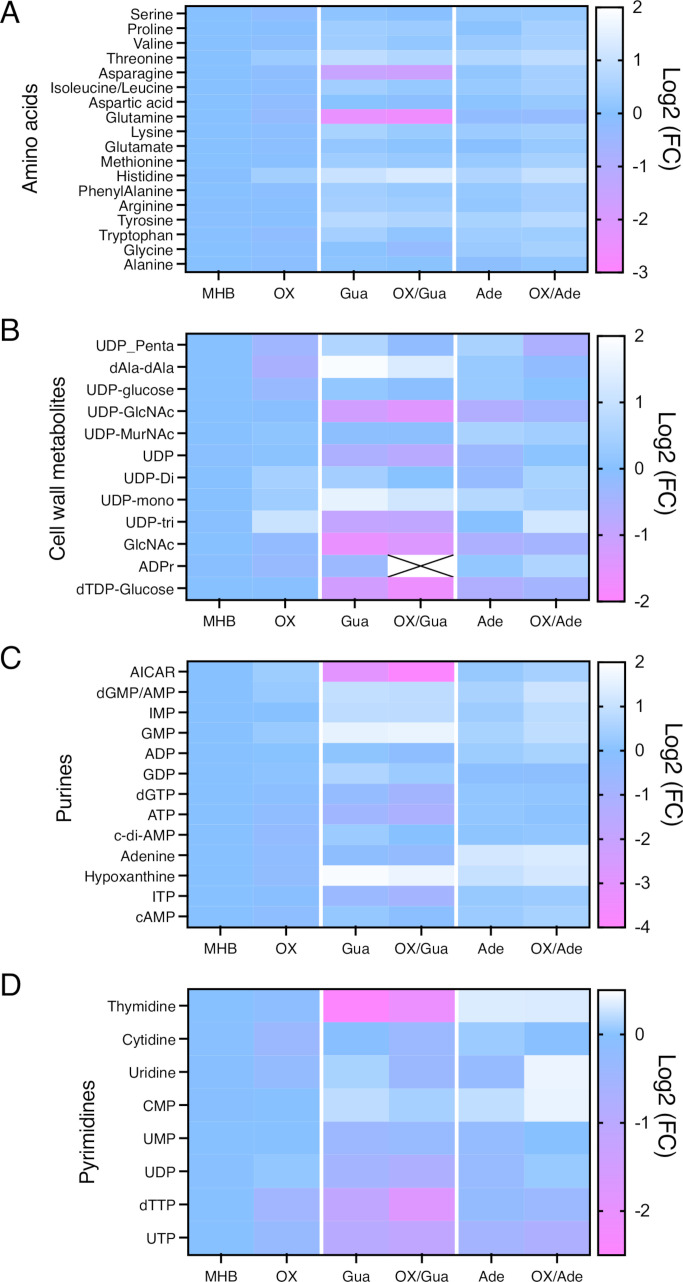
Exposure of MRSA to guanosine (Gua) or adenosine (Ade) has pleiotropic effects on the MRSA metabolome. Heatmap comparison of amino acids (**A**), cell wall metabolites (**B**), purines (**C**), and pyrimidines (**D**) in JE2 grown in Mueller-Hinton Broth (MHB) and MHB supplemented with Gua (200 µg/mL) or Ade (200 µg/mL) with or without oxacillin (OX, 1 µg/mL). The data presented are the average of three biological replicates analyzed using GraphPad Prism V9. Individual metabolite levels that were significantly different were identified using a one-way analysis of variance.

To further understand the mechanistic basis for altered β-lactam susceptibility in MRSA exposed to Gua or Ade, intracellular metabolite levels were compared in cultures supplemented with either nucleoside alone or in combination with OX (1 µg/mL; Fig. 1; Fig. S1). As expected, exposure to Gua or Ade was accompanied by significant intracellular accumulation of each nucleoside (Fig. S1) and pleiotropic effects on the metabolome (Fig. 1).

**Fig 2:**
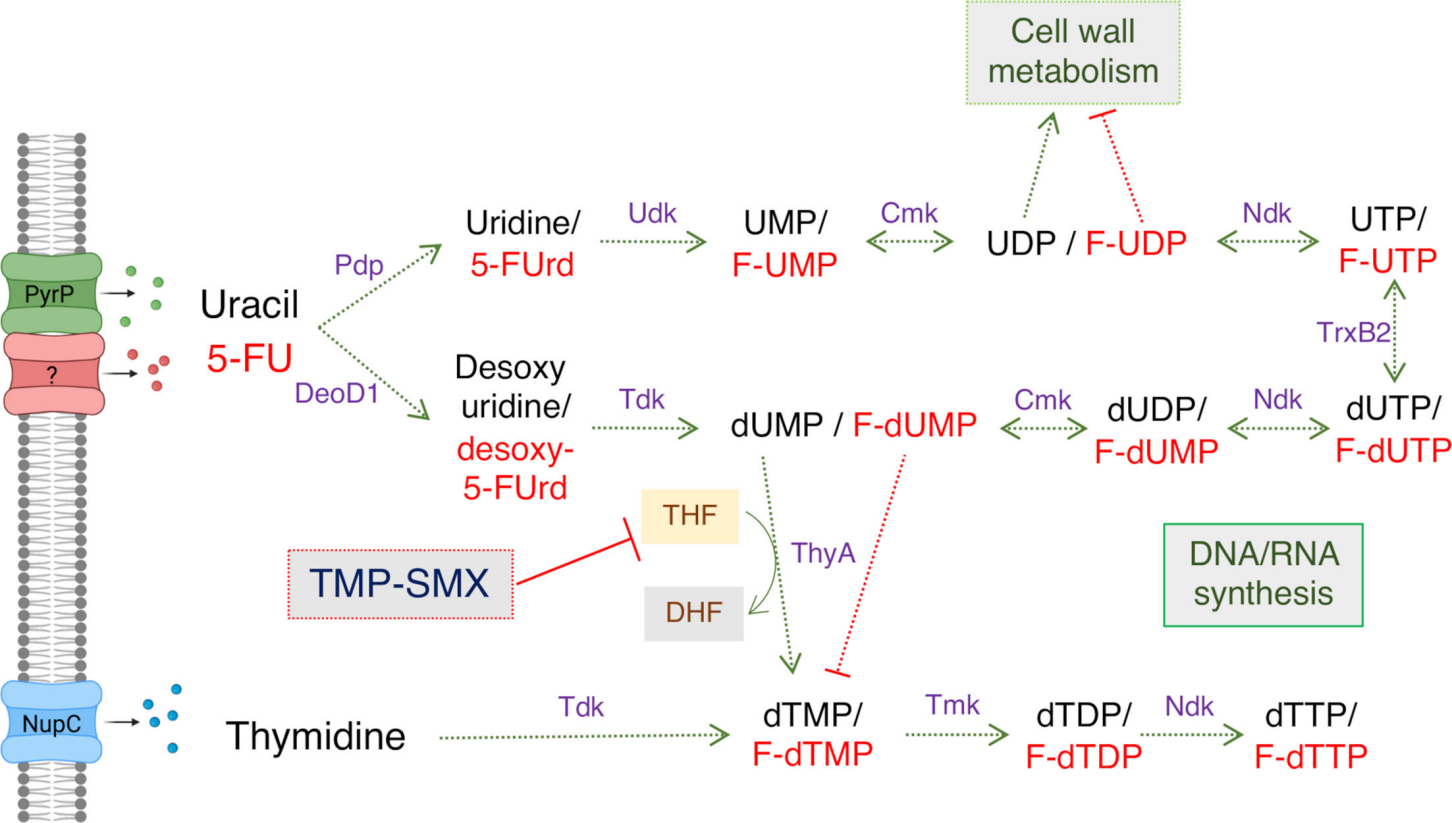
Overview of thymidine, uracil, and 5-fluorouracil (5-FU) transport and metabolism, including enzymes and intermediates implicated in susceptibility to 5-FU and trimethoprim-sulfamethoxazole (TMP-SMX). The permease PyrP (also known as UraA) has previously been implicated in the transport of uracil (11, 12), but the transporter for 5-FU in *S. aureus* has not yet been identified. Uracil is converted to uridine and deoxyuridine by Pdp and DeoD1, respectively, before being fluxed to UMP/dUMP, UDP/dUTP, and UTP/dUTP. dUMP is converted to dTMP by thymidylate synthase (ThyA) (13) in a reaction requiring tetrahydrofolic acid (THF) as a co-factor, which is converted to dihydrofolic acid (DHF). UDP is an important intermediate in cell wall metabolism, which is adversely affected by the antimetabolite 5-F-UDP. 5-FU exposure leads to the buildup of F-UMP and F-TMP, which interfere with the normal incorporation of UMP and TMP into DNA and RNA, respectively. TMP-SMX inhibits two enzymatic reactions in the folate pathway, thereby interfering with the synthesis of THF required for ThyA activity (7). Transport of exogenous thymidine by NupC (7) can bypass ThyA-dependent dTMP production. Figure created using biorender.com.

Among the most notable changes were significant Gua-induced reductions in the levels of the purine biosynthetic intermediate AICAR, several cell wall precursors, the amino acids asparagine and glutamine, and the pyrimidine nucleotide thymidine (Fig. 1). Asparagine serves as a precursor for purine biosynthesis (9), and its depletion may contribute to, or be a consequence of, disrupted purine homeostasis in MRSA treated with Gua. Glutamine is an important NH_4_^+^ donor for the amidation of iso-d-glutamate in the peptidoglycan stem pentapeptide (10). Furthermore, glutamine is also an essential substrate for pyrimidine biosynthesis, and its depletion in MRSA exposed to Gua may contribute to reduced levels of thymidine. The significant reduction in thymidine levels raised the possibility that Gua may also potentiate the activity of antibiotics, including anti-folates, that interfere with pyrimidine and/or thymidine biosynthesis (Fig. 2).

### Anti-MRSA activity of oxacillin combined with 5-fluorouracil or antifolates is significantly increased by guanosine

The impact of the Gua-induced reduction in thymidine levels on MRSA susceptibility to the anti-folate antibiotics TMP-SMX and the pyrimidine analog drug 5-FU was investigated. 5-FU disrupts pyrimidine metabolism and has previously been reported to cause “thymidine-less death” in bacteria (14). Exogenous 5-FU is taken up and converted into fluorinated analogs of uridine/deoxyuridine, namely 5-fluorouridine/5-fluorodeoxyuridine (5-FUrd/deoxy 5-FUrd) (14–16) (Fig. 2). Checkerboard assays revealed significant synergy between Gua and 5-FU or 5-FUrd against JE2, but not with TMP, SMX, or TMP-SMX (Table 1). Combinations of OX with 5-FU, 5-FUrd, and TMP were also synergistic (Table 1). Strikingly, Gua (200 µg/mL) potentiated the activity of combinations of these antibiotics, as evidenced by lower ΣFIC values, including OX/SMX and OX/TMP-SMX that on their own were not synergistic (Table 1).

**TABLE 1:** Antibacterial activity (MIC) and drug synergy (fractional inhibitory concentration indices, ΣFIC) of oxacillin (OX), 5-fluorouracil (5-FU), 5-fluorouridine (5-FUrd), trimethoprim (TMP), sulfamethoxazole (SMX), and TMP-SMX in combination with and without guanosine (Gua, 200 µg/mL), against MRSA strain JE2

Single antibiotic	MIC (µg/mL)^*a*^		
OX	64		
5-FU	32		
5-FUrd	0.06		
TMP	1		
SMX	128–256		
TMP-SMX	0.25		

Single antibiotic + Gua	MIC (µg/mL)^*b*^		ΣFIC^*c*^
OX/Gua	2–4		0.03
5-FU/Gua	16		0.5
5-FUrd/Gua	0.03		0.5
TMP/Gua	1		1
SMX/Gua	128		1
TMP-SMX/Gua	0.5		1

Double antibiotic combinations	MICs (µg/mL)		ΣFIC
OX/5-FU	OX 8	5-FU 8	0.25
OX/5-FUrd	OX 0.25	5-FUrd 0.012	0.2
OX/TMP	OX 0.25	TMP 0.25	0.25
OX/SMX	OX 16	SMX 64	0.75
OX/TMP-SMX	OX 32	TMP-SMX 0.12	1

Double antibiotic + Gua combinations	MICs (µg/mL)^*d*^		ΣFIC
OX/5-FU/Gua	OX 0.5	5-FU 0.5	0.016
OX/5-FUrd/Gua	OX < 0.06	5-FUrd < 0.003	<0.05
OX/TMP/Gua	OX 0.25	TMP 0.12	0.12
OX/SMX/Gua	OX 0.5	SMX 8	0.03
OX/TMP-SMX/Gua	OX 1	TMP-SMX 0.03	0.12

^*a*^ MIC, minimum inhibitory concentration; µg/mL.

^*b*^ MIC of the antibiotic in the presence of Gua or another antibiotic; µg/mL.

^*c*^ FIC indices (ΣFIC) for antibiotic/Gua, antibiotic/antibiotic, and antibiotic/antibiotic + Gua combinations. The fractional inhibitory concentration (FIC) is the MIC of the antibiotic in the presence of Gua or another antibiotic divided by the MIC of the antibiotic alone); mg/mL. The ΣFIC = FIC A + FIC B, where FIC A is the MIC of the first antibiotic in combination with Gua or another antibiotic/MIC of the first antibiotic alone, and FIC B is the MIC of the second antibiotic in combination with Gua or another antibiotic/MIC of the second antibiotic alone. The combination is considered synergistic when the ΣFIC is ≤0.5 (highlighted by pale green shading), indifferent when the ΣFIC is >0.5 to <2 (highlighted by pale orange shading).

^*d*^ When two antibiotics were used in combination with each other in the presence of Gua, the FIC of each antibiotic in the presence of both the second antibiotic and Gua is indicated.

**Fig 3:**
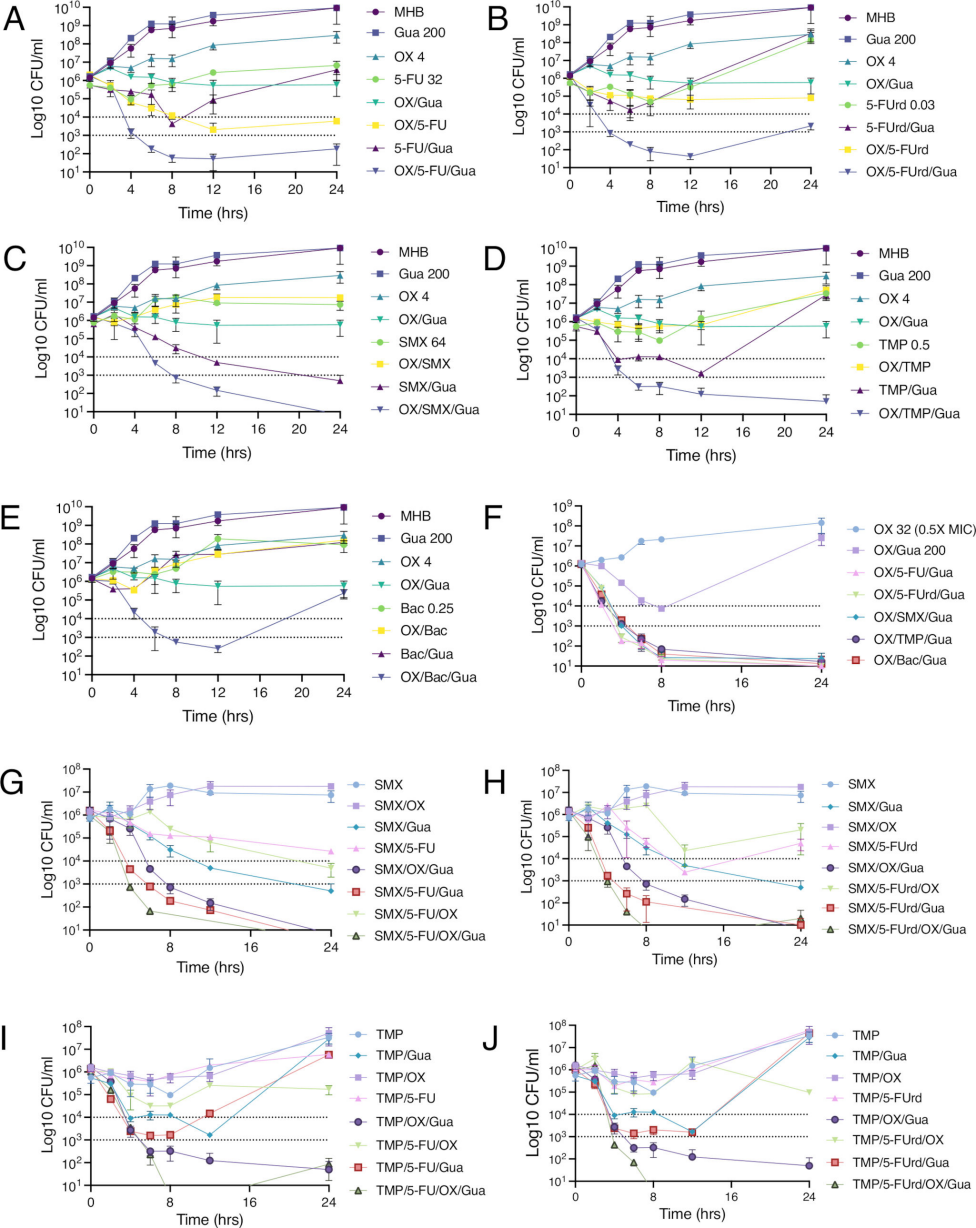
Guanosine (Gua) potentiates MRSA killing by oxacillin (OX), anti-folate antibiotics, and pyrimidine analogs. Time-kill assays were performed using 0.5 × MICs of 5-fluorouracil (5-FU; 32 µg/mL), 5-fluorouridine (5-FUrd; 0.03 µg/mL), sulfamethoxazole (SMX; 64 µg/mL), trimethoprim (TMP; 0.5 µg/mL), or Bactrim (SMX-TMP; 0.25 µg/mL), alone or in combination with Gua (200 µg/mL) and/or OX (4 µg/mL [0.0625 × MIC] or 32 µg/mL [0.5 × MIC]). Panels show killing kinetics for: (**A**) 5-FU ± OX/Gua; (**B**) 5-FUrd ± OX/Gua; (**C**) SMX ± OX/Gua; (**D**) TMP ± OX/Gua; (**E**) Bactrim ± OX/Gua; (**F**) 0.5 × OX ± Gua or Gua/antimetabolite combinations; (**G**) SMX ± OX, 5-FU, and Gua; (**H**) SMX ± OX, 5-FUrd, and Gua; (**I**) TMP ± OX, 5-FU, and Gua; and (**J**) TMP ± OX, 5-FUrd, and Gua. Exponential phase cultures were inoculated into Mueller-Hinton Broth (MHB) 2% NaCl at a starting cell density of approximately 1 × 10^6^ CFU/mL, with or without antibiotics and Gua as indicated, and CFUs were enumerated after 0, 2, 4, 6, 8, 12, and 24 h. The limit of detection in these experiments is 10^1^ CFU/mL. The data presented are the average of three independent experiments plotted using GraphPad Prism V9, and standard deviations are shown.

Time-kill assays used to investigate potential bactericidal activity further revealed significant synergy (>2 log reduction in the number of JE2 colony-forming units [CFUs]/mL) between Gua and 5-FU, 5-FUrd, SMX, and TMP (0.5 × minimum inhibitory concentration [MICs]; Fig. 3A through D), but not TMP-SMX (Fig. 3E). Notably, bactericidal activity (>3 log CFU/mL reduction) was achieved when low dose OX (4 µg/mL; 0.0625 × MIC) was added to these antibiotic/Gua combinations (Fig. 3A through E). Further increasing the OX concentration to 32 µg/mL (0.5 × MIC) achieved a near eradication of JE2 after 24 h for all 5-FU, 5-FUrd, SMX, TMP, and TMP-SMX combinations (Fig. 3F). Finally, the most significant bactericidal activity was measured when Gua was included with triple SMX/5-FUrd/OX, TMP/5-FU/OX, TMP/5-FUrd/OX, and SMX/5-FU/OX combinations, which achieved eradication or near eradication of JE2 after 8–16 h (Fig. 3G through J).

Using Nebraska Transposon Mutant Library mutants implicated in nucleotide transport and metabolism revealed that the reversal of 5-FU- and 5-FUrd-induced JE2 growth inhibition by exogenous thymidine or uridine, respectively (Fig. S2), was *nupC*-dependent. NupC was previously reported as the primary thymidine transporter in *S. aureus* (7).

Extending these analyses to other MRSA strains, disk diffusion assays revealed that Gua also potentiated the activity of 5-FU and 5-FUrd, but not SMX or TMP, against MW2, COL, and BH1CC (Table S1). However, double-disk diffusion assays with SMX and TMP also revealed that synergy between these two anti-folates against MW2, COL, and BH1CC was enhanced by Gua (Fig. S3; Table S1). Furthermore, the anti-MRSA activity of combinations of these antimicrobial drugs was further enhanced by Gua for all strains (the only exception was SMX-5-FU/Gua against MW2; Table S1).

Taken together, these findings support an important role for the downregulation of thymidine in the antibiotic adjuvant activity of Gua. Gua potentiates the anti-MRSA activity of anti-folate and pyrimidine analog drugs, as well as β-lactams. Combinations of these antibiotics with Gua can eradicate MRSA, albeit in a strain-dependent manner.

### Eradication of MRSA biofilms by antibiotic/guanosine combinations

Antibiotics were tested at concentrations approximating their reported maximum serum levels (*C*_max_): OX (200 µg/mL) (17–19), SMX (78 µg/mL) and TMP (4 µg/mL) (20), 5-FU (68 µg/mL) (18), 5-FUrd (200 µg/mL) (21), and vancomycin (VAN, 50 µg/mL) (22), which is used to treat MRSA infections, was included as a positive control. Each antibiotic was evaluated alone and in combination with Gua (200 µg/mL), which is not reported to be toxic (23).

**Fig 4:**
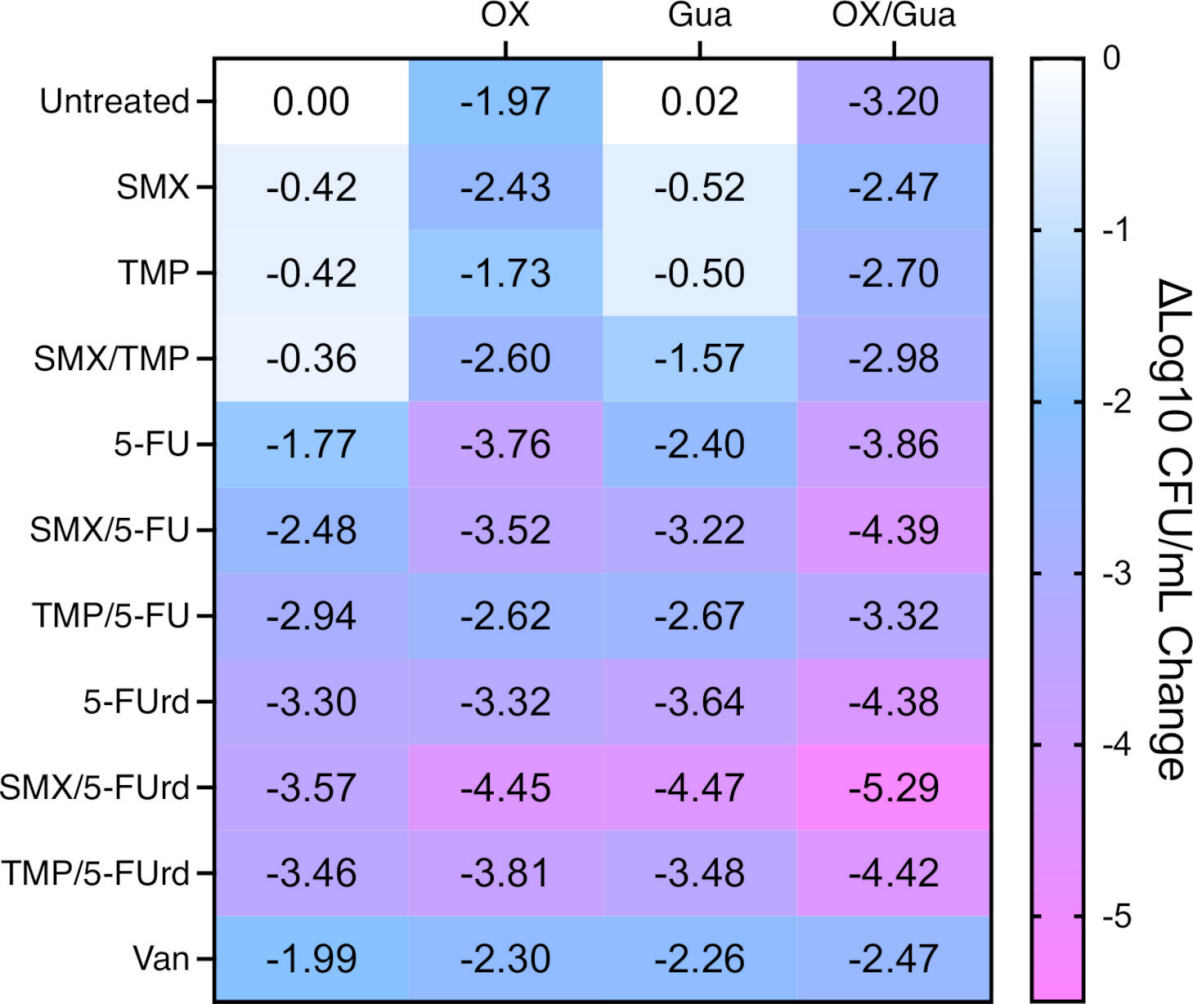
Oxacillin/guanosine combinations with anti-folates and pyrimidine analogs enhance JE2 biofilm eradication. Pre-formed 24 h JE2 biofilms were treated for an additional 24 h with antibiotics or the adjuvant guanosine singly and in combinations at concentrations approximating reported *C*_max_ values. Surviving cells were serially diluted, enumerated on Mueller-Hinton Agar (MHA), and the results presented as Δlog₁₀ CFU/mL relative to untreated controls. The antibiotic/adjuvant concentrations used were: OX, 200 μg/mL; SMX, 78 μg/mL; TMP, 4 μg/mL; 5-FU, 68 μg/mL; 5-FUrd, 200 μg/mL; VAN, 50 μg/mL; and Gua, 200 μg/mL. The data, which represent the mean of three independent experiments, were plotted using GraphPad Prism v9.

Exposure of pre-formed JE2 biofilms to OX alone produced a ~2 log reduction in CFU/mL, comparable to VAN. The OX/Gua combination further improved activity, achieving approximately threefold reduction (Fig. 4). TMP and SMX, individually or combined with Gua, showed minimal anti-biofilm activity. In contrast, 5-FU and 5-FUrd were more effective, particularly when paired with Gua (Fig. 4). Strikingly, combinations of OX with TMP, SMX, 5-FU, or 5-FUrd yielded 2–4 log reductions in CFU/mL (Fig. 4). Although adding Gua did not enhance the activity of OX/5-FU or OX/5-FUrd, both OX/5-FUrd and OX/5-FUrd/Gua achieved 4–5 log reductions, significantly greater than the 2-log reduction observed with VAN (Fig. 3) and surpassing the 1–3 log reductions typically reported for single antimicrobial agents (24). These 4–5 log reductions are in line with accepted thresholds for disinfection and are comparable to the most effective anti-biofilm antimicrobial combinations reported to date (24–26).

### Guanosine exacerbates cell envelope stress induced by oxacillin and 5-fluorouracil

**Fig 5:**
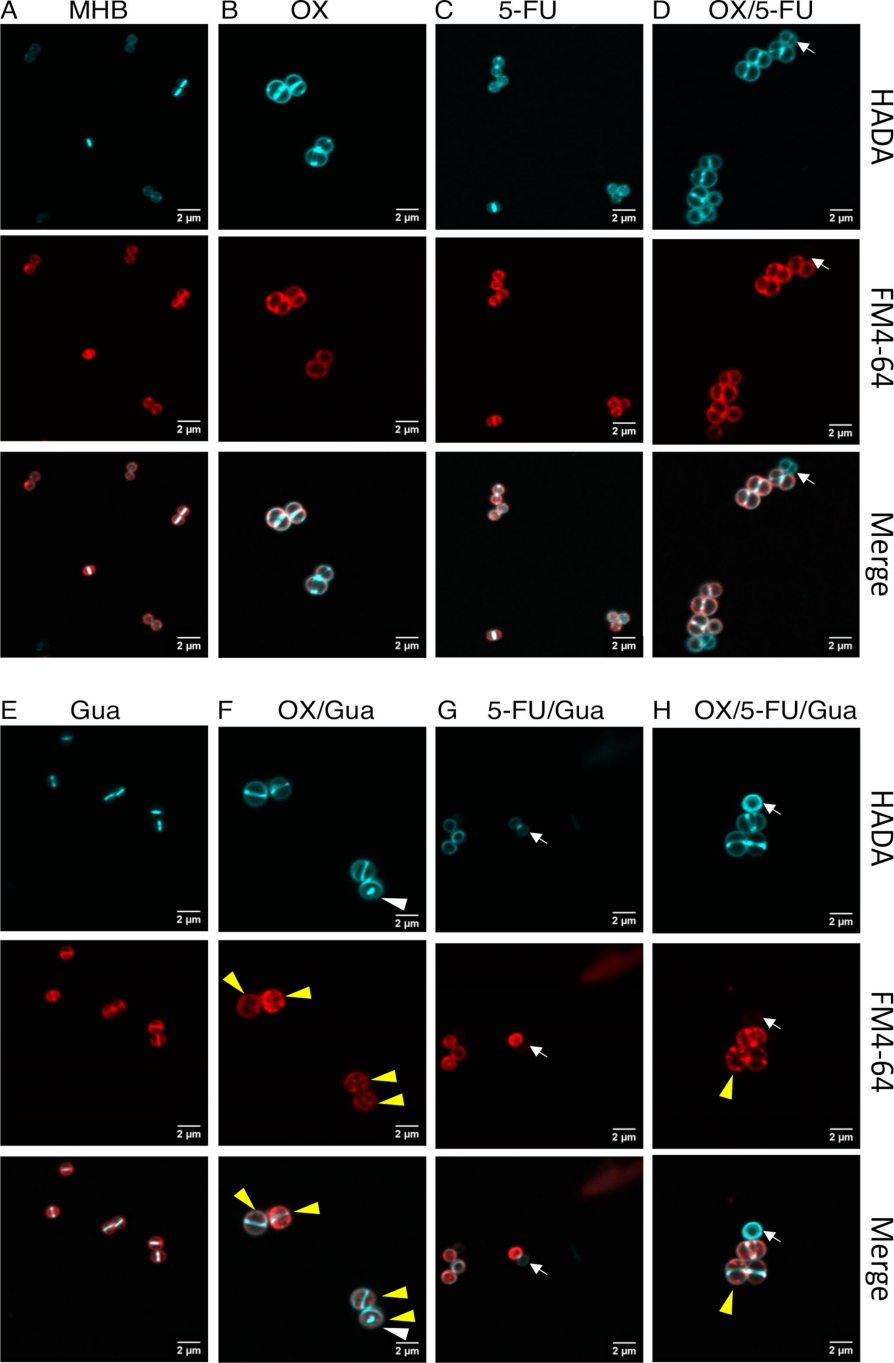
Guanosine (Gua) potentiates oxacillin (OX)/5-fluorouracil (5-FU)-induced perturbation of the MRSA cell envelope. Representative confocal microscopy images of JE2 cells grown in: (**A**) MHB; (**B**) MHB + OX (1 µg/mL); (**C**) MHB + Gua (200 µg/mL); (**D**) MHB + OX/Gua; (**E**) MHB + 5-FU (1 µg/mL); (**F**) MHB + 5-FU/Gua; (**G**) MHB + OX/5-FU; (**H**) MHB + OX/5-FU/Gua. Cells were stained with HADA to label newly synthesized peptidoglycan and FM4-64 to visualize *S. aureus* membranes. White arrows highlight cells that incorporated HADA but did not take up FM4-64. Yellow triangles mark cells with membrane dye aggregates, and white triangles indicate cells exhibiting HADA-labeled peptidoglycan clumps. Images were acquired at 1,000× magnification using an Olympus LS FLUOVIEW FV3000 confocal laser scanning microscope and processed with ImageJ (Fiji v1.0).

Confocal microscopy imaging of JE2 exposed to OX, 5-FU, and Gua, alone and in combinations, was used to probe the mechanism(s) of growth inhibition and cell killing (Fig. 5). Consistent with previous reports (1, 27–30), OX-treated cells were enlarged with characteristic membrane bulges at sites perpendicular to the septum (Fig. 5A and B). Peptidoglycan incorporation visualized with HADA (31, 32) was localized to the septum in Mueller-Hinton Broth (MHB)- and OX-grown cells, which also exhibited uniform staining with the membrane dye FM4-64 (33) (Fig. 5A and B; Fig. S4). Subinhibitory 5-FU altered HADA labeling (Fig. 5C; Fig. S4), previously associated with weakened cell wall integrity (34). Some OX/5-FU-treated cells incorporated HADA while failing to incorporate FM4-64 (Fig. 5D; Fig. S4), suggestive of altered membrane dye uptake despite normal peptidoglycan staining. Cells treated with Gua alone were similar to MHB-grown cells (Fig. 5E), but a small proportion did not take up the HADA dye (Fig. S4). OX/Gua-treated cells exhibited prominent HADA- and FM4-64-stained intracellular clumps that were absent from cells treated with OX alone (Fig. 5F), with a higher proportion of cells stained only with HADA or FM4-64 (Fig. S4). Staining of cells treated with 5-FU/Gua was similar to 5-FU alone (Fig. 5G; Fig. S4). Finally, the triple combination (OX/5-FU/Gua) increased the proportion of cells lacking FM4-64 or HADA staining altogether and was accompanied by extensive membrane clumping and multiple HADA-labeled division sites (Fig. 5H; Fig. S4). Taken together, these observations show that while OX and 5-FU individually perturb distinct aspects of the cell envelope, the addition of Gua intensifies these defects, generating cells with inconsistent membrane dye uptake, aberrant peptidoglycan accumulation, and multiple or stalled division sites. The combined OX/5-FU/Gua treatment therefore exerts pleiotropic and synergistic effects on both membrane integrity and cell wall synthesis, consistent with extensive cell envelope stress.

### Guanosine exacerbates ROS accumulation and disrupts membrane potential in combination with oxacillin and 5-fluorouracil

**Fig 6:**
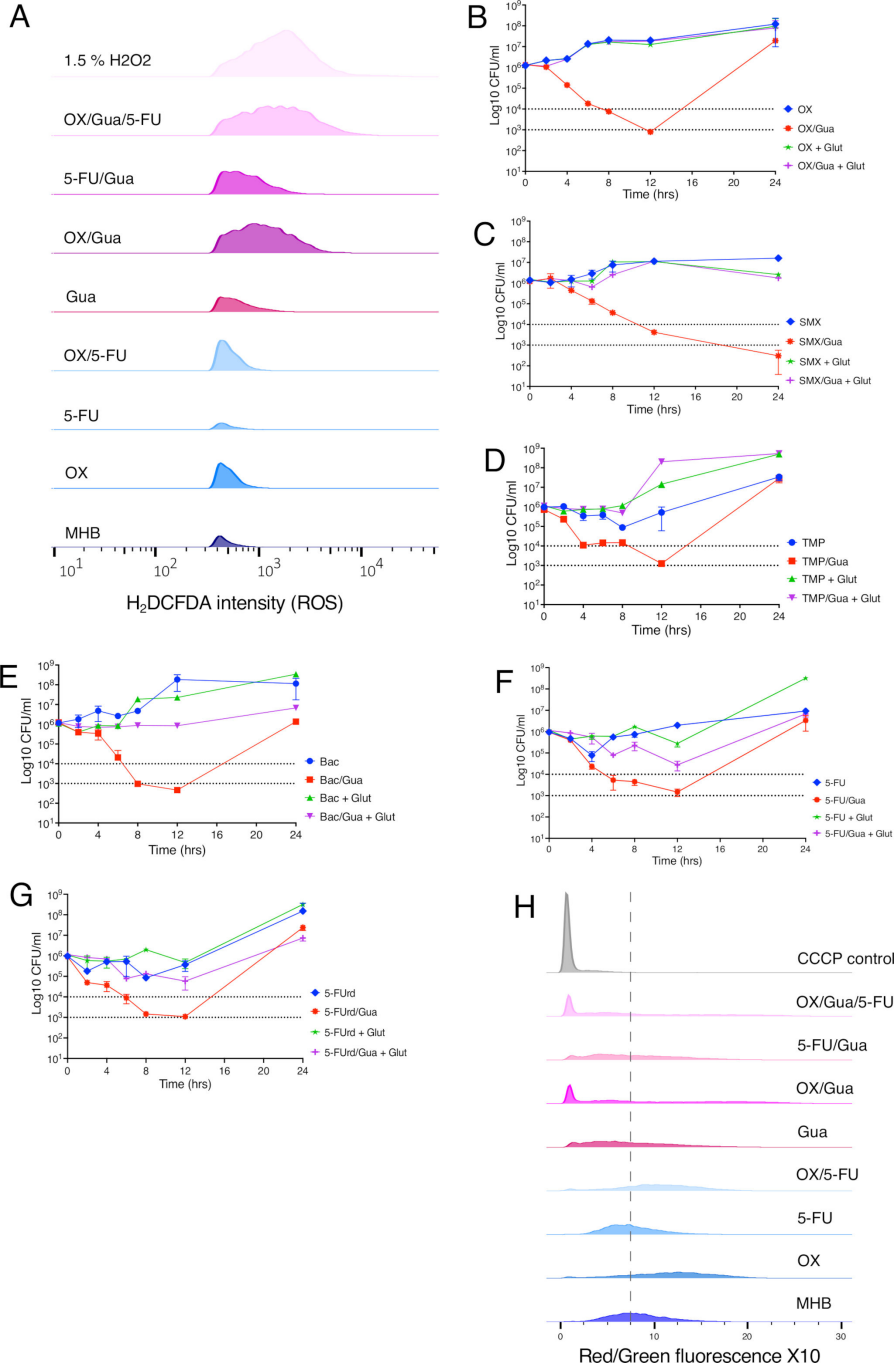
Guanosine (Gua) enhances antibiotic-induced oxidative stress and membrane depolarization in MRSA. (**A**) Intracellular ROS levels measured using the fluorescent probe carboxy-H2DCFDA. JE2 cells were grown to the exponential phase and exposed to Gua, oxacillin (OX), 5-fluorouracil (5-FU), or their combinations, then stained with carboxy-H2DCFDA (10 µM) for 10 min. A 1.5% H_2_O_2_ treatment served as a positive control and produced robust fluorescence. Histograms show relative fluorescence versus cell counts. Data were processed using FlowJo v10.1, and one biological replicate from three independent experiments is shown. (**B–G**) Time-kill assays comparing the impact of guanosine and antioxidant supplementation on antibiotic-mediated killing. Exponential phase cultures were treated with OX (32 µg/mL), sulfamethoxazole (SMX, 64 µg/mL), trimethoprim (TMP, 0.5 µg/mL), Bactrim (Bac, 0.25 µg/mL), 5-FU (32 µg/mL), 5-fluorouridine (5-FUrd, 0.06 µg/mL) alone or in combination with Gua (200 µg/mL), and/or glutathione (Glut, 10 mM). Panels show killing kinetics for: (**B**) OX ± Gua alone or Gua + Glut; (**C**) SMX ± Gua alone or Gua + Glut; (**D**) TMP ± Gua alone or Gua + Glut; (**E**) Bac ± Gua alone or Gua + Glut; (**F**) 5-FU ± Gua alone or Gua + Glut; and (**G**) 5-FUrd ± Gua alone or Gua + Glut. Exponential phase cultures were inoculated into MHB 2% NaCl at a starting cell density of approximately 1 × 10^6^ CFU/mL, with or without antibiotics and Gua as indicated, and CFUs were enumerated after 0, 2, 4, 6, 8, 12, and 24 h. The limit of detection in these experiments is 10^1^ CFU/mL. The data presented are the average of three independent experiments plotted using GraphPad Prism V9, and standard deviations are shown. (**H**) Membrane potential measurements using DiOC2(3) and flow cytometry. Exponential-phase JE2 cells were treated with Gua, OX, 5-FU, or their combinations, stained with DiOC2(3), and analyzed by FACS. Carbonyl cyanide m-chlorophenylhydrazone (CCCP; 15 µM final concentration) was used as a control to collapse the membrane potential. For each sample, 10,000 gated events were collected. Red/green fluorescence ratios × 10 were plotted as histograms. Data were processed using FlowJo v10.1, and one biological replicate from four independent experiments is shown.

ROS contribute to the mechanism of action for compounds targeting thymidine biosynthesis, including SMX-TMP (35–37). ROS accumulation may, in turn, impact membrane integrity as described earlier (Fig. 6). ROS levels were quantified using H_2_DCFDA, a fluorogenic probe activated by intracellular oxidation (38). Cells were grown to the exponential phase, treated with OX, Gua, 5-FU, or their combinations, and analyzed by flow cytometry alongside an H_2_O_2_ control. OX treatment increased ROS levels (Fig. 6A), consistent with β-lactam-induced ROS accumulation (39). ROS levels in cells treated with OX/5-FU were similar to OX alone, whereas 5-FU alone had little effect. Gua-treated cells also showed elevated ROS. Strikingly, OX/Gua and OX/Gua/5-FU combinations produced ROS levels comparable to the strong inducer H_2_O_2_ (40), indicating severe oxidative stress under these conditions. In killing assays, the ROS scavenger glutathione (10 mM) (41) reversed the bactericidal activity of antibiotics (OX, SMX, TMP, Bac, 5-FU, and 5-FUrd)/Gua combinations (Fig. 6B through G), supporting the importance of ROS in the adjuvant activity of Gua. Glutathione alone did not affect MRSA killing by any of these antibiotics (Fig. 6B through G). Notably, Gua enhanced the bactericidal activity of Bac in MHB (Fig. 6E), an effect not observed in MHB supplemented with NaCl (Fig. 3E), indicating that NaCl interferes with the synergy between Gua and Bac.

Membrane potential was assessed using the fluorescent dye DiOC2(3) (42). Increased red:green fluorescence ratios indicate elevated membrane potential, whereas decreased ratios reflect dissipation of the proton motive force (PMF), a hallmark of cell death (43, 44). OX increased the red:green ratio, while 5-FU had minimal effect and OX/5-FU resembled OX-treated cells (Fig. 6H). In contrast, Gua-treated cells displayed a reduced red:green ratio, indicating membrane depolarization. Importantly, Gua/OX and Gua/OX/5-FU treatments were accompanied by a pronounced shift toward low red:green ratios, comparable to carbonyl cyanide m-chlorophenylhydrazone (CCCP)-treated cells, indicative of a substantial PMF collapse that may contribute to reduced viability (Fig. 6H). Together, these data indicate that Gua-induced ROS and membrane depolarization, which are amplified in combination with OX (and to a lesser extent 5-FU), are likely to contribute to extensive cell envelope defects, growth inhibition, and cell killing.

## DISCUSSION

Building on our recent report that the purine nucleosides guanosine and xanthosine increase MRSA susceptibility to β-lactam antibiotics (1), we report here that exposure to guanosine significantly reduces thymidine levels and increases susceptibility to the anti-folate antibiotics SMX and TMP, as well as the pyrimidine analogs 5-FU and 5-FUrd. SMX and TMP are cost-effective antibiotics often used in combination for the treatment of MRSA infections. These drugs target sequential enzymes in folate synthesis that feed into thymidine production (45–47), but their effectiveness is undermined by resistance associated with mutations in *dfrB* and *thyA* (8, 48–50). While the efficacy of SMX or TMP depends on thymidine levels (47) and growth inhibition of JE2 on blood agar plates supplemented with TMP was previously shown to be thymidine dependent (13), adaptive mutations associated with reduced central metabolism also contribute to *S. aureus* survival of SMX-TMP treatment (51). Our data reveal potent anti-MRSA activity of either SMX or TMP in combination with both Gua and OX. This novel drug combination may also limit the emergence of SMX-TMP-resistant MRSA strains and be effective against MRSA strains resistant to non-β-lactam antimicrobial drugs.

Reductions in the levels of the purine intermediate AICAR, glutamine, and thymidine were among the most striking observations from metabolomic analysis of MRSA exposed to guanosine. Interestingly, levels of the pyrimidine nucleotide cytidine were not significantly reduced by Gua. The major impact of guanosine on nucleotide homeostasis makes it challenging to speculate on why glutamine and thymidine levels in particular are significantly downregulated. Because thymidine, but not cytidine, biosynthesis depends on THF, the reduced levels of thymidine in Gua-treated cells are indicative of impaired folate metabolism. GTP is the substrate for the first committed step of folate biosynthesis: the FolE/FolE2-catalyzed conversion of GTP to dihydroneopterin triphosphate (52, 53). Elevated GMP levels (Fig. S2) in Gua-treated cells may also interfere with normal levels of purine biosynthesis feedback inhibition, thereby impacting purine/GTP-dependent THF availability and thymidine biosynthesis. Reduced levels of several peptidoglycan precursors in Gua-treated cells may also be a consequence of reduced glutamine availability and contribute to enhanced β-lactam susceptibility. MRSA cells treated with guanosine and oxacillin also displayed altered membrane labeling using FM4-64, an observation that may also be consistent with downregulation of peptidoglycan synthesis enzymes and β-lactam resistance (54–56).

The data presented here indicate that reduced thymidine in OX/Gua-treated MRSA exposes an enhanced vulnerability to pyrimidine antimetabolites (5-FU and 5-FUrd) that target thymidine synthesis (57). Guanosine-mediated depletion of thymidine also potentiates the activity of SMX, TMP, and the SMX-TMP combination when used with oxacillin, revealing an alternative treatment strategy for infections typically managed with SMX-TMP alone, particularly where therapeutic failures or suboptimal responses arise (8, 58). For example, SMX-TMP has relatively weak activity against MRSA biofilms (59), whereas the guanosine/oxacillin/5-FU or 5-FUrd combinations described in this study also have significant anti-biofilm activity, raising the possibility that they could be used to improve the management of device-related and other chronic infections. 5-FU has previously been reported to interfere with LuxS/autoinducer-2-regulated biofilm in *S. aureus* (60). The antibacterial properties of fluorinated pyrimidine antagonists, which are used in the treatment of cancer over longer time periods, indicate their potential clinical usefulness as antimicrobial drugs (61, 62). Indeed, 5-FU and 5-FUrd have been used successfully to treat staphylococcal infections in mice (63, 64). The absence of cross resistance between 5-FU/5-FUrd and SMX/TMP further paves the way to their deployment in the treatment of infections caused by TMP-SMX-resistant *S. aureus* (65).

The consequences of the guanosine-induced reduction in thymidine levels were evident in both increased ROS generation and dissipated membrane potential. ROS is a known contributor to the mechanism of action of compounds such as SMX-TMP that reduce thymidine levels and is accompanied by DNA damage and cell death (35, 36). Levels of ROS in MRSA exposed to guanosine or guanosine/oxacillin/5-FU combinations were comparable to those induced by 1.5% H_2_O_2_. Supporting a role for oxidative stress in bactericidal activity, the ROS scavenger glutathione attenuated MRSA killing by antibiotic/guanosine combinations. Increased ROS levels were also accompanied by dissipation of membrane potential in cells treated with oxacillin and guanosine, suggesting that oxidative damage compromises proton motive force and membrane energetics. ROS-driven collapse of membrane potential has previously been linked to bacterial cell death (66) and may also contribute to enhanced MRSA killing.

Together, these data reveal a novel therapeutic approach using the adjuvant guanosine to re-sensitize MRSA to β-lactams and clinically used anti-folate or pyrimidine-based drugs, supporting the development of combination therapies that improve the treatment options for MRSA infections without requiring new classes of antimicrobial drugs.

## MATERIALS AND METHODS

### Bacterial strains and growth conditions

Bacterial strains are listed in Table S2. All strains were grown in MHB or Mueller-Hinton Agar (MHA), supplemented with Gua, Ade, and 5-5-FU where indicated.

### Comparative metabolomic analysis

Intracellular metabolite analysis was performed as described previously (67, 68). A total of 250 mL flasks containing 25 mL MHB (with or without 1 μg/mL OX, 200 µg/mL Gua, or 200 µg/mL Ade) were inoculated from 5 mL overnight cultures at a starting OD_600_ = 0.06 and grown for 4–5 h. Culture volumes corresponding to OD_600_ = 10 were harvested and rapidly filtered through a 0.45 µm membrane (Millipore). The cells on the membrane were washed twice with 5 mL cold saline and immediately quenched in ice-cold 60% ethanol containing 2 µM Br-ATP and 2 µM Ribitol as internal controls. The cells were mechanically disrupted using a bead homogenizer set to oscillate for 3 × 30 s cycles at 6,800 rpm with a 10 s pause between each cycle. Cell debris was separated by centrifugation at 12,000 rpm. Supernatants containing intracellular metabolites were lyophilized and stored at −80°C. These samples were reconstituted in 100 µL of 50% MeOH. LC-MS/MS analysis was carried out as described previously (69).

### Antibiotic MIC measurements and synergy/checkerboard assays

MIC measurements by broth microdilutions were performed in accordance with CLSI methods for dilution susceptibility testing of staphylococci (70) with modifications as follows: guanosine, xanthosine, or adenosine were supplemented into culture media at a final concentration of 200 µg/mL. Strains were first grown at 37°C on MHA 2% NaCl for 24 h and 5–10 colonies were resuspended in 0.85% saline before being adjusted to 0.5 McFarland standard (OD_600_ = 0.1). The cell suspensions were then diluted 1:20 in PBS and 10 µL used to inoculate 100 µL media (MHB 2% NaCl/MHB 2% NaCl with 200 µg/mL guanosine) containing serially diluted antibiotics (oxacillin, 5-fluorouracil, 5-fluorouridine, sulfamethoxazole, and trimethoprim) as indicated. The plates were incubated at 37°C for 24 h, and MIC values were recorded as the lowest antibiotic concentration where no growth was observed. Checkerboard/synergy assays performed as previously described (1), utilized 96-well plates in which one antibiotic/adjuvant was serially diluted vertically and the second antibiotic was serially diluted horizontally.

### Disc diffusion synergy assays

Disc diffusions were carried out according to CLSI guidelines with the following modifications (71). Strains were grown overnight in TSB, serially diluted 1:100 (in fresh TSB), and grown for 4 h at 37°C at 200 rpm. The cultures were then adjusted to OD_600_ = 0.5 and spread evenly across 4 mm thick MHA plates. Discs impregnated with antimicrobial drugs were placed onto plates and allowed to grow for 20 h. To measure synergy, two discs were placed 15 mm apart and grown for 20 h to determine if there was increased growth inhibition in the region between the disks on MHA or MHA Gua (200 μg/mL).

### Antibiotic killing assays

Antibiotic killing assays were performed according to CLSI guidelines (70), as previously described (1). Briefly, overnight cultures grown in MHB were diluted 1:100 and grown for 3 h before being inoculated into 25 mL of MHB with or without antibiotics as indicated in 250 mL flasks at a starting cell density of approximately 1 × 10^6^ CFU/mL. For experiments measuring oxacillin susceptibility, the overnight cultures were grown in MHB 2% NaCl, and the experiments were performed in MHB 2% NaCl. The flasks were incubated at 35°C with shaking at 200 rpm, and CFUs were enumerated on TSA plates after 0, 2, 4, 6, 8, 12, and 24 h. Glutathione (10 mM final concentration) was added as indicated.

### Biofilm eradication assays

Biofilm eradication assays were performed as described previously (72). Briefly, biofilms were grown in 2 mL brain heart infusion medium (BHI) inoculated into the wells of 24-well tissue culture coated plates at 37°C for 24 h before being carefully washed twice with PBS. BHI supplemented with antibiotics as indicated was then added to the biofilm wells, and the biofilms were incubated for a further 24 h at 37°C. The biofilms were dispersed by scraping, serially diluted, and CFUs were enumerated on TSA plates. The antibiotic concentrations used in these experiments were equal to or less than the *C*_max_ in humans as follows: oxacillin 200 µg/mL (17), sulfamethoxazole 78 µg/mL, trimethoprim 4 µg/mL (20), 5-fluorouracil 68 µg/mL (18), 5-fluorouridine 200 µg/mL (21), and vancomycin 50 µg/mL (22). An upper *in vivo* toxicity limit for guanosine has not been reported (23), and 200 µg/mL was used in this study. Three biological replicates were performed for each condition.

### Confocal microscopy using FM4-64 and HADA

For imaging of membranes and newly synthesized peptidoglycan, JE2 overnight cultures were inoculated 1:100 into 5 mL MHB cultures or MHB supplemented with oxacillin (1 µg/mL), 5-fluorouracil (1 µg/mL), and/or guanosine (200 µg/mL) as indicated. These cultures were then grown for 3 h before being incubated with HADA (500 µM final concentration) in the dark for 5 min. Next, the cells were pelleted for 2 min at 14,000 × *g* and resuspended in PBS supplemented with +2 µg/mL FM4-64 at a cell density of OD_600_ = 1 before being incubated for 20 min at 37°C. The cells were then washed twice with 1 mL PBS before being resuspended in PBS (OD_600_ = 1), and 5 µL aliquots were spot inoculated onto a 1% PBS agarose pad. The stained bacteria were then imaged at 1,000× magnification using an Olympus LS FLUOVIEW Fv3000 Confocal Laser Scanning Microscope. Images were analyzed using Fiji (ImageJ) software, and representative images are shown. FM4-64 or HADA accumulation was quantified using Fiji in cells (*n* = 90) treated with antibiotics alone and in combination with guanosine compared to cells grown in MHB across three biological samples and plotted using GraphPad Prism v9.

### Antibiotic susceptibility spot dilution assays

Overnight cultures were adjusted to OD_600_ of 1 in PBS, and 10-fold serial dilutions were made in a 96-well plate (20 µL of more concentrated suspension into 180 µL PBS). A total of 5 µL of each dilution was spot inoculated onto MHA and MHA containing antibiotics ± guanosine (200 µg/mL) ± thymidine (50 μg/mL).

### Membrane potential measurements

The membrane potential of JE2 grown under different conditions was measured using 3,3′-diethyloxacarbocyanine iodide (DiOC2(3)) dye using flow cytometry, using a previously described method (40, 42) with modifications. JE2 overnight cultures were inoculated 1:100 into 5 mL MHB or MHB supplemented with oxacillin (1 µg/mL), 5-fluorouracil (1 μg/mL), and/or guanosine (200 µg/mL) as indicated. These cultures were grown to OD_600_ = 0.8 before 1 mL aliquots were harvested by centrifugation at 14,000 rpm, and the cells were washed twice with 1 mL of PBS. Thereafter, the cells were diluted to OD_600_ = 0.2 in PBS, and a 100 µL of cell suspension aliquot was added to 890 µL PBS and DiOC2(3) dye (30 µM final concentration). CCCP (15 µM final concentration) was used as a control to collapse the membrane potential. A total of 10,000 gated events were recorded per sample using BD Accuri C6 Plus Sampler Flow cytometer and BD Accuri C6 Plus Software, using the FL1 (green) and FL3 (red) channels. These experiments were performed on four biological replicates, and the averages and standard deviation were plotted using FlowJo software V10.1, as described previously (40).

### ROS quantification

Intracellular ROS were quantified using H_2_DCFDA; this probe is taken up by living cells and oxidized by intracellular ROS, producing a fluorescent readout (38). JE2 overnight cultures were inoculated 1:100 into 5 mL MHB or MHB supplemented with oxacillin (1 µg/mL), 5-fluorouracil (1 μg/mL), and/or guanosine (200 μg/mL) as indicated. These cultures were grown to OD_600_ = 0.8, and the cell density was adjusted to OD_600_ = 0.2 in 1 mL volume before being stained with H_2_DCFDA fluorescent dye (final concentration 10 µM) as described previously (73). The cell suspensions were incubated for 10 min before 800 µL PBS supplemented with 100 mM EDTA was added to 200 µL of stained cells. This suspension was dispensed into flow tubes and wrapped in aluminum foil. ROS levels were measured by BD Accuri C6 Plus Sampler Flow cytometer and BD Accuri C6 Plus Software using the FL-1 channel, and 10,000 gated events (cells) were recorded. The data were analyzed using FlowJo software V10.1.

## SUPPLEMENTAL MATERIAL

Fig. S1 aac.00377-26-s0001.tif Supplemental figure 1.

Fig. S2 aac.00377-26-s0002.tif Supplemental figure 2.

Fig. S3 aac.00377-26-s0003.tif Supplemental figure 3.

Fig. S4 aac.00377-26-s0004.tif Supplemental figure 4.

Table S1 aac.00377-26-s0005.docx Antibacterial activity and synergy of cloxacillin, sulfamethoxazole, trimethoprim, 5-fluorouracil, 5-fluorouridine, and Bactrim, alone and in combination with guanosine, against MRSA strains JE2, MW2, COL, and BH1CC.

Table S2 aac.00377-26-s0006.docx Bacterial strains and plasmids used in this study.

ASM does not own the copyrights to Supplemental Material that may be linked to, or accessed through, an article. The authors have granted ASM a non-exclusive, world-wide license to publish the Supplemental Material files. Please contact the corresponding author directly for reuse.
