# Supplementary figures and images for: The β-lactam adjuvant guanosine potentiates anti-folate antibiotics and pyrimidine synthesis inhibitors by depleting thymidine in methicillin-resistant Staphylococcus aureus
Source: Antimicrob Agents Chemother. 2026 Jun 10;70(7):e00377-26. doi: 10.1128/aac.00377-26 (PMC13321833; doi:10.1128/aac.00377-26)

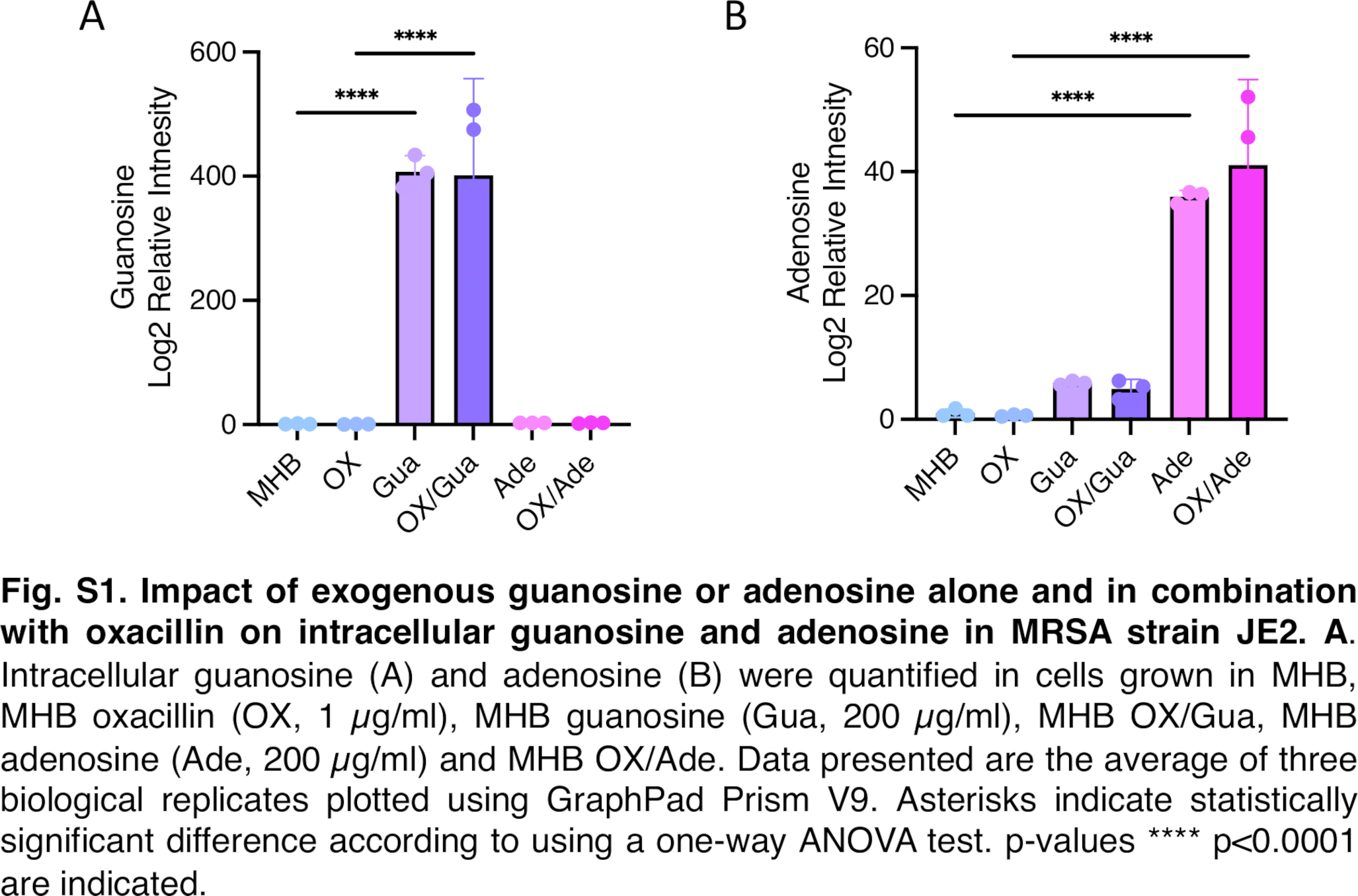

Supplement: Fig. S1 — Supplemental figure 1. [file aac.00377-26-s0001.tif]

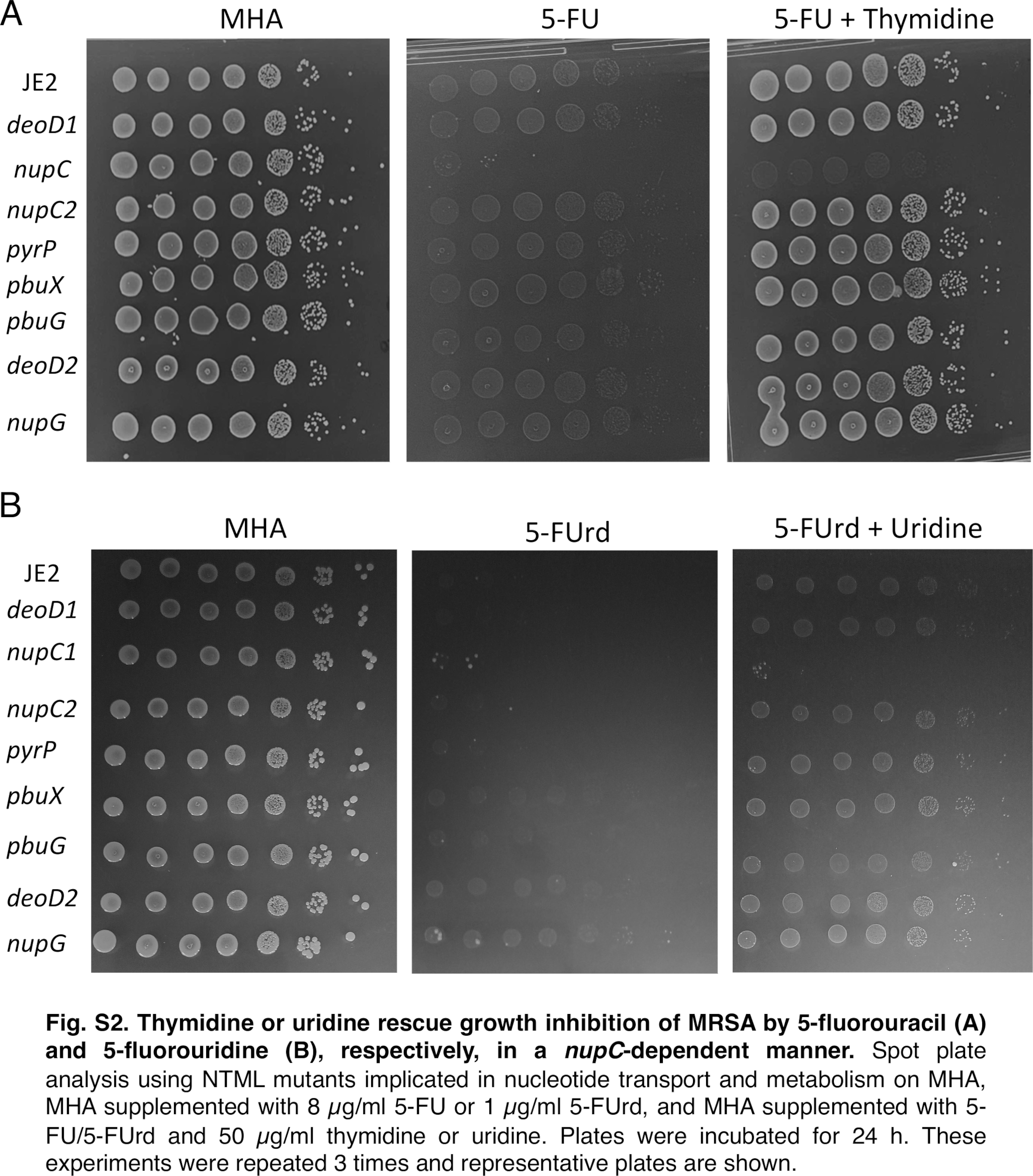

Supplement: Fig. S2 — Supplemental figure 2. [file aac.00377-26-s0002.tif]

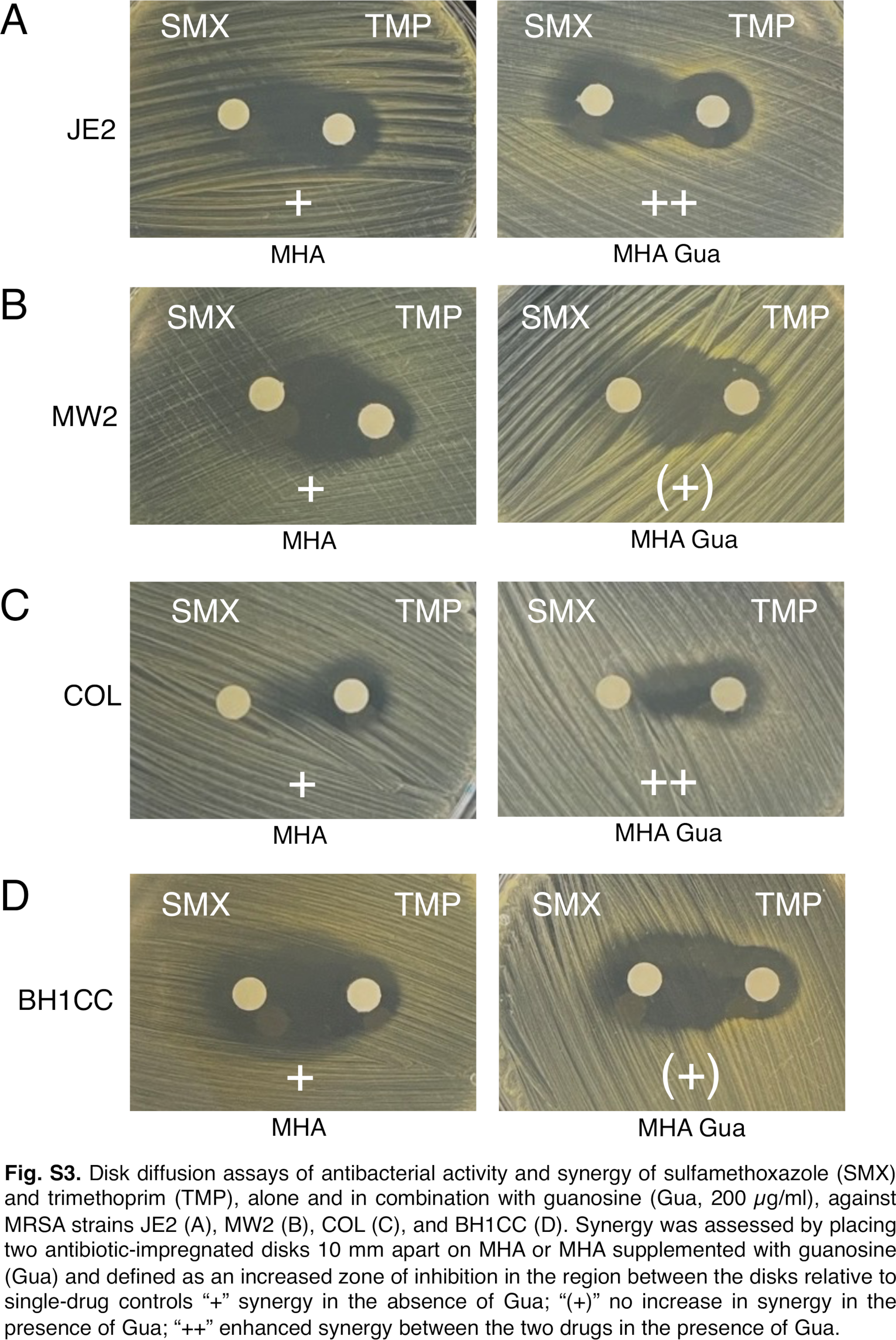

Supplement: Fig. S3 — Supplemental figure 3. [file aac.00377-26-s0003.tif]

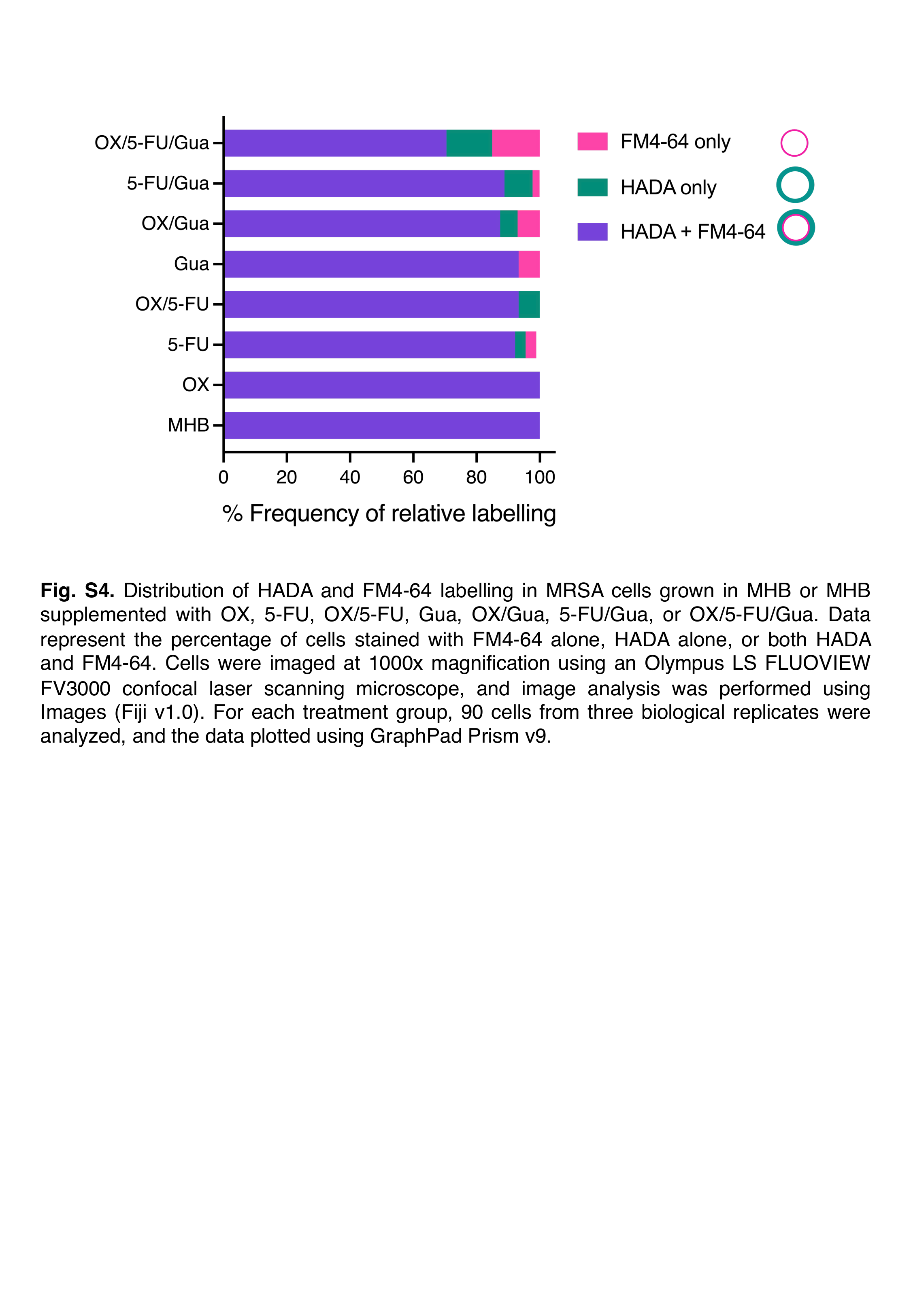

Supplement: Fig. S4 — Supplemental figure 4. [file aac.00377-26-s0004.tif]
