# Supplementary material for: The β-lactam adjuvant guanosine potentiates anti-folate antibiotics and pyrimidine synthesis inhibitors by depleting thymidine in methicillin-resistant Staphylococcus aureus
Source: Antimicrob Agents Chemother. 2026 Jun 10;70(7):e00377-26. doi: 10.1128/aac.00377-26 (PMC13321833; doi:10.1128/aac.00377-26)
Supplement: Table S1 — Antibacterial activity and synergy of cloxacillin, sulfamethoxazole, trimethoprim, 5-fluorouracil, 5-fluorouridine, and Bactrim, alone and in combination with guanosine, against MRSA strains JE2, MW2, COL, and BH1CC. [file aac.00377-26-s0005.docx]

**Table S1.** Antibacterial activity (zone diameters, mm) and synergy of cloxacillin (Clox), sulfamethoxazole (SMX), trimethoprim (TMP), 5-fluorouracil (5-FU), 5-fluorouridine (5-FUrd), and Bactrim (Bac), alone and in combination with guanosine (Gua, 200 µg/ml), against MRSA strains JE2, MW2, COL, and BH1CC.

**Strain**

**Drug +/- Gua**

|  | JE2 | MW2 | COL | BH1CC |
| --- | --- | --- | --- | --- |
| Clox | 11 | 6 | 6 | 6 |
| Clox/Gua | 29 | 12 | 6 | 9 |
| SMX | 6 | 6 | 6 | 6 |
| SMX/Gua | 15 | 6 | 6 | 14 |
| TMP | 19 | 18 | 17 | 19 |
| TMP/Gua | 20 | 20 | 21 | 21 |
| 5-FU | 14 | 6 | 11 | 19 |
| 5-FU/Gua | 17 | 12 | 25 | 19 |
| 5-FUrd | 15 | 11 | 16 | 16 |
| 5-FUrd/Gua | 16 | 17 | 24 | 20 |
| Double drug combinations +/- Gua^1^ | | | | |
| SMX-TMP | + | + | + | + |
| SMX-TMP/Gua | ++ | (+) | ++ | (+) |
| Clox-5-FU | + | - | + | - |
| Clox-5-FU/Gua | ++ | ++ | ++ | ++ |
| Clox-5-FUrd | + | + | - | - |
| Clox-5-FUrd/Gua | ++ | ++ | ++ | ++ |
| SMX-5-FU | + | - | - | - |
| SMX-FU/Gua | ++ | - | ++ | ++ |
| SMX-5-FUrd | + | - | - | - |
| SMX-5-FUrd/Gua | ++ | ++ | ++ | ++ |
| TMP-5-FU | + | - | + | + |
| TMP-5-FU/Gua | (+) | ++ | ++ | ++ |
| TMP-5-FUrd | + | + | + | + |
| TMP-5-FU/Gua | (+) | ++ | ++ | ++ |

^1^ Synergy was assessed by placing two antibiotic-impregnated disks 10 mm apart on MHA plates and defined as an increased zone of inhibition in the region between the disks relative to single-drug controls. “-“ no detectable synergy; “+” synergy in the absence of Gua; “(+)” no increase in synergy in the presence of Gua; “++” enhanced synergy between the two drugs in the presence of Gua.
