# Supplementary material for: The β-lactam adjuvant guanosine potentiates anti-folate antibiotics and pyrimidine synthesis inhibitors by depleting thymidine in methicillin-resistant Staphylococcus aureus
Source: Antimicrob Agents Chemother. 2026 Jun 10;70(7):e00377-26. doi: 10.1128/aac.00377-26 (PMC13321833; doi:10.1128/aac.00377-26)
Supplement: Table S2 — Bacterial strains and plasmids used in this study. [file aac.00377-26-s0006.docx]

**Table S2.** Bacterial strains and plasmids used in this study

| **Strains** | **Relevant Details** |
| --- | --- |
| JE2 | USA300 cured of p01 & p03. Parent of Nebraska Transposon Mutant Library (NTML). |
| MW2 | MRSA SCCmec type IV; CC1 (1) |
| COL | MRSA reference strain; SCC*mec* type I; CC8 (2) |
| BH1CC | MRSA clinical isolate; SCC*mec* type II; CC8 (3) |
| NE1419 *nupG* | JE2 *nupG* (SAUSA300_0611). Erm^r^. (4) |
| NE283 *pbuG/stgP* | JE2 *pbuG/stgP* (SAUSA300_2207). Erm^r^. (4) |
| NE280 *pbuX* | JE2 *pbuX* (SAUSA300_0387). Erm^r^. (4) |
| NE650 *deoD2* | JE2 *deoD2* (SAUSA300_2091). Erm^r^. (4) |
| NE477 *deoD1* | JE2 *deoD1* (SAUSA300_0138). Erm^r^. (4) |
| NE544 *nupC1* | JE2 *nupC1* (SAUSA300_0298). Erm^r^. (4) |
| NE622 *nupC2* | JE2 *nupC2* (SAUSA300_0313). Erm^r^. (4) |
| NE1048 *pyrP* | JE2 *pyrP* (SAUSA300_1092). Erm^r^. (4) |

**References**

1. **Saiman L, O'Keefe M, Graham PL, 3rd, Wu F, Said-Salim B, Kreiswirth B, LaSala A, Schlievert PM, Della-Latta P.** 2003. Hospital transmission of community-acquired methicillin-resistant *Staphylococcus aureus* among postpartum women. Clin Infect Dis **37:**1313-1319.

2. **Gill SR, Fouts DE, Archer GL, Mongodin EF, Deboy RT, Ravel J, Paulsen IT, Kolonay JF, Brinkac L, Beanan M, Dodson RJ, Daugherty SC, Madupu R, Angiuoli SV, Durkin AS, Haft DH, Vamathevan J, Khouri H, Utterback T, Lee C, Dimitrov G, Jiang L, Qin H, Weidman J, Tran K, Kang K, Hance IR, Nelson KE, Fraser CM.** 2005. Insights on evolution of virulence and resistance from the complete genome analysis of an early methicillin-resistant *Staphylococcus aureus* strain and a biofilm-producing methicillin-resistant *Staphylococcus epidermidis* strain. J Bacteriol **187:**2426-2438.

3. **O'Neill E, Pozzi C, Houston P, Smyth D, Humphreys H, Robinson DA, O'Gara JP.** 2007. Association between methicillin susceptibility and biofilm regulation in *Staphylococcus aureus* isolates from device-related infections. J Clin Microbiol **45:**1379-1388.

4. **Fey PD, Endres JL, Yajjala VK, Widhelm TJ, Boissy RJ, Bose JL, Bayles KW.** 2013. A genetic resource for rapid and comprehensive phenotype screening of nonessential *Staphylococcus aureus* genes. mBio **4:**e00537-00512.
